# Lung cancer screening by low-dose computed tomography: a cost-effectiveness analysis of alternative programmes in the UK using a newly developed natural history-based economic model

**DOI:** 10.1186/s41512-020-00087-y

**Published:** 2020-12-02

**Authors:** Edward Griffin, Chris Hyde, Linda Long, Jo Varley-Campbell, Helen Coelho, Sophie Robinson, Tristan Snowsill

**Affiliations:** 1 Peninsula Technology Assessment Group (PenTAG), College of Medicine and Health, University of Exeter, St Luke’s campus, Heavitree Road, Exeter, EX1 2LU UK; 2 Exeter Test Group, College of Medicine and Health, University of Exeter, St Luke’s campus, Heavitree Road, Exeter, EX1 2LU UK; 3 Health Economics Group, College of Medicine and Health, University of Exeter, St Luke’s campus, Heavitree Road, Exeter, EX1 2LU UK

**Keywords:** Screening, Lung cancer, Diagnosis, Low-dose computed tomography, Natural history model, Cost-effectiveness, QALY

## Abstract

**Background:**

A systematic review of economic evaluations for lung cancer identified no economic models of the UK setting based on disease natural history. We first sought to develop a new model of natural history for population screening, then sought to explore the cost-effectiveness of multiple alternative potential programmes.

**Methods:**

An individual patient model (ENaBL) was constructed in MS Excel® and calibrated against data from the US National Lung Screening Trial. Costs were taken from the UK Lung Cancer Screening Trial and took the perspective of the NHS and PSS. Simulants were current or former smokers aged between 55 and 80 years and so at a higher risk of lung cancer relative to the general population. Subgroups were defined by further restricting age and risk of lung cancer as predicted by patient self-questionnaire. Programme designs were single, triple, annual and biennial arrangements of LDCT screens, thereby examining number and interval length. Forty-eight distinct screening strategies were compared to the current practice of no screening. The primary outcome was incremental cost-effectiveness of strategies (additional cost per QALY gained).

**Results:**

LDCT screening is predicted to bring forward the stage distribution at diagnosis and reduce lung cancer mortality, with decreases versus no screening ranging from 4.2 to 7.7% depending on screen frequency. Overall healthcare costs are predicted to increase; treatment cost savings from earlier detection are outweighed by the costs of over-diagnosis. Single-screen programmes for people 55–75 or 60–75 years with ≥ 3% predicted lung cancer risk may be cost-effective at the £30,000 per QALY threshold (respective ICERs of £28,784 and £28,169 per QALY gained). Annual and biennial screening programmes were not predicted to be cost-effective at any cost-effectiveness threshold.

**Limitations:**

LDCT performance was unaffected by lung cancer type, stage or location and the impact of a national screening programme of smoking behaviour was not included.

**Conclusion:**

Lung cancer screening may not be cost-effective at the threshold of £20,000 per QALY commonly used in the UK but may be cost-effective at the higher threshold of £30,000 per QALY.

**Supplementary Information:**

**Supplementary information** accompanies this paper at 10.1186/s41512-020-00087-y.

## Highlights

- ENaBL is a new economic model based on the observed natural history of lung cancer and calibrated to the US National lung screening trial.
- Additional advantages of evaluating multiple populations and programme designs and adjusting for lead time bias, length bias and over-diagnosis.
- LDCT lung screening is predicted to be effective in reducing lung cancer mortality relative to no screening in the range 4.2 to 7.7% depending on screen frequency.
- Programmes are shown to increase diagnoses, including over-diagnosis, and increase costs.
- LDCT screening is unlikely to be cost-effective at the £20,000/QALY threshold, but a single screen of 60–75 year olds with ≥ 3% risk may be cost-effective at £30,000/QALY.

## Background

Lung cancer is a continuing major global public health problem, and in the UK it is the leading cause of cancer death (22%) [1]. In 2013, the UK had an above EU average death rate of 61.6 deaths per 100,000 [2]. Approximately 46,400 cases of lung cancer were diagnosed in 2014, representing 13% of the total number of cancer cases [1]. The prognosis for long-term survival is poor. One-year survival for adults in England and Wales in 2010–2011 was 32.1%. Cancer Research UK estimated 5- and 10-year survival in 2010/2011 to be 9.5 and 4.9%, respectively, in England and Wales [3].

Although the potential effectiveness of screening with low-dose computed tomography (LDCT) has been demonstrated in large trials like the National Lung Screening Trial (NLST) in the USA, there is unresolved uncertainty about cost-effectiveness [4]. A systematic review of existing economic evaluations of LDCT screening for lung cancer has revealed markedly varied estimates. Few reviews commented on the generalisability of their findings, but certain assumptions regularly appeared as significant in determining cost-effectiveness. Important factors are the cost of a LDCT scan; the risk of lung cancer in the screened cohort (pertaining to prevalence but also incidence for studies evaluating more than a single screen); the effectiveness of LDCT screening in broad terms, for example, achieving a stage shift without significant over-diagnosis; extending lung cancer survival beyond lead time; and reducing lung cancer mortality. Two economic evaluations identified in the review were conducted in the UK setting, based on the UK Lung Cancer Screening Trial (UKLS) [5–7]. Both concluded that LDCT screening could be cost-effective in the UK. The latter of these evaluations included a comparison with an economic evaluation based on the NLST, highlighting the likely reasons why they found a disparity [8, 9]. A third, recently published, UK evaluation was based on findings from a community-based LDCT pilot and a reconstruction of the UKLS model [10]. However, these UK-based economic evaluations have not been based on the highest quality evidence (although they have produced somewhat consistent results in terms of incremental QALYs compared with studies that are based on high-quality evidence), have not tested multiple screening programmes or populations and have not predicted the natural history of lung cancer in the absence of screening. Outside the UK, three different natural history models have been used to predict the cost-effectiveness of LDCT screening. The Lung Cancer Policy Model suggested that LDCT in the USA would not be cost-effective [11]. The Cancer Risk Management Model (renamed OncoSim) suggested that biennial LDCT in the Canadian setting would be cost-effective [12, 13]. Finally, the Microsimulation Screening Analysis (MISCAN) lung model suggested that annual LDCT, also in Canada, would be cost-effective [14]. We aimed, therefore, to firstly develop a new model—able to simulate occult disease—and secondly examine the cost-effectiveness of multiple alternative potential screening programme formats in the UK setting. This independent economic model, called the Exeter NAtural history-Based economic model of Lung cancer screening (ENaBL), takes our understanding of screening in the UK setting further. It is parametrised using high-quality evidence and adjusts for the positive biases associated with screen-detected cases, which may all inflate the cost-effectiveness of screening. These are lead time bias, which may artificially inflate screening effectiveness because earlier diagnosis may not prolong survival in cases when death is not delayed by earlier intervention; length bias, which tends to increase in the proportion of slower progressing less aggressive tumours, the detection of which may not translate to greater survival; and over-diagnosis, because tumours detected by screening that would not have clinically presented prior to death from other causes are frequently attributed health benefits. ENaBL is able to mitigate for these biases and also assesses multiple screening programmes across alternative populations defined by age, gender and risk score, enabling decision-makers to better evaluate different policy/programme options. This paper and associated monograph (for full methodological detail) conform to the Consolidated Health Economic Evaluation Reporting Standards (CHEERS) [15, 16].

## Methods

ENaBL is an individual patient simulation model developed using a discrete event simulation framework whereby individual patients were sampled according to baseline age, sex, presence of preclinical disease and predicted minimum risk of disease. This approach allows transparent and flexible modelling of known patient and disease heterogeneity and the time dependency of related events. Individual outcomes were simulated across four alternative screening programmes defined by the number of LDCT screening rounds and the time between rounds:

- (S) Single one-off screen shortly following entry (UKLS protocol) [5]
- (T) Triple screen comprising a first screen shortly following entry and subsequent screens at 12 and 24 months (NLST protocol) [4]
- (B) Biennial repeated screening from shortly after entry then 24 monthly from entry date but not beyond 80th birthday
- (A), as described in (B) but screens are repeated annually (USPSTF recommendations) [17].

The target population was current or former smokers within the aged range 55 and 80 years with a higher risk of lung cancer relative to the general population, informed by a survey of an external advisory group. Twelve population groups were defined by combinations of further age restriction (55–80, 60–80, 55–75 and 60–75 years) and alternative minimum threshold of predicted risk (≥ 3%, ≥ 4% and ≥ 5%) as calculated using the Liverpool Lung Project tool version 2 [18, 19]. Individuals meeting the criteria in the population group were selected from a pool of 20,000 simulated individuals for invitation into a screening strategy (costs and QALYs were very stable after 15,000 simulations). Individuals not meeting the criteria or failing to partake were retained and received no screening (control). In total, there were 48 intervention strategies.

The evaluation setting was the NHS in the UK, and the cost perspective was the NHS and personal social services (PSS). Only direct health effects on individuals were included, no attempt has been made to model societal impacts such as modified smoking behaviour [20]. The primary outcome was the incremental cost-effectiveness ratios between strategies, expressed in additional cost per QALY gained (£, 2016/2017). Secondary outcomes included screening programme sensitivity; relative risk of lung cancer diagnosis, probability of early diagnosis versus late, average lead time, number of lung cancers diagnosed per 100,000 entrants (including interval cancers), reduction in mortality due to lung cancer, 5-year lung cancer survival and change in age at diagnosis/death from lung cancer.

A lifetime time horizon was used in order to capture all relevant costs and benefits, discounted at 3.5% per year as per UK standard. Costs, QALYs and other outcomes for each strategy were estimated using a decision tree which identified appropriate simulated individuals from the pool and assigned them to either one or more screening interventions (if they meet all criteria and wished to join the screening programme) or to no screening.

**Fig. 1:**
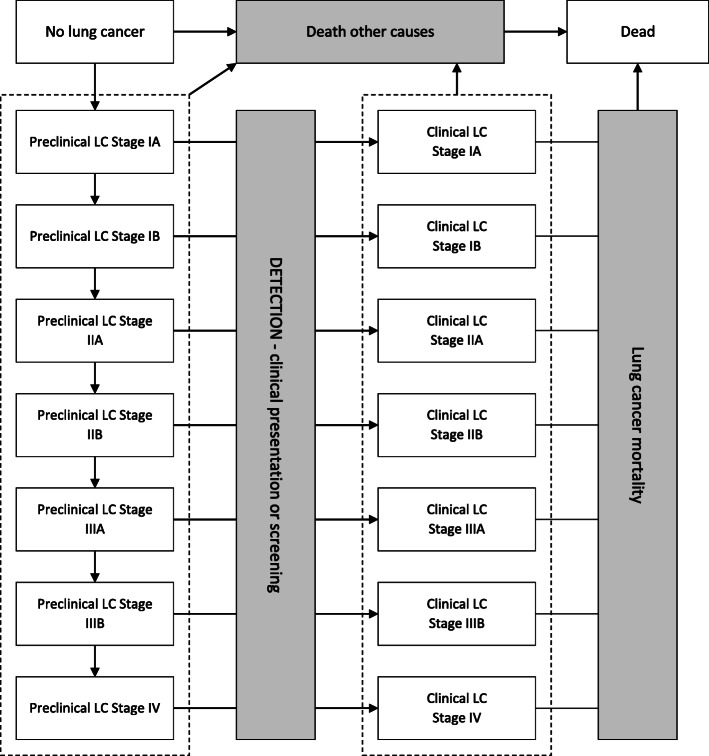
Model diagram for simulating individuals. LC, lung cancer. White boxes represent health states; grey boxes and arrows represent events. An individual begins the simulation without clinically diagnosed lung cancer, because they do not have lung cancer or they have occult lung cancer (health states on the left hand side). He/she is immediately at risk of incidental development of lung cancer, preclinical progression of the occult tumour, detection or death from other causes. It is assumed that death from lung cancer is preceded by a diagnosis. Following detection, the individual is again at risk of progression or death, now from either from lung cancer or other causes

In all strategies, individuals begin the simulation without clinically diagnosed lung cancer, although they could have preclinical (occult) lung cancer. A natural history model was developed to generate disease incidence and progression for individuals in the absence of screen detection. In the event of a screening round, at a pre-determined time since programme enrolment, participants were either diagnosed or cleared to continue in the programme. There was no explicit modelling of cancer progression after diagnosis: output costs and outcomes are intended to be averaged across lung cancers diagnosed in each stage. A diagram of possible patient flow through the model is depicted in Fig. 1.

The rate of lung cancer detection by screening gives rise to the differences in health benefit in each strategy (earlier transition through ‘DETECTION’ in Fig 1). If detected cancers were at an earlier stage than they would have presented clinically, then the time to lung cancer mortality was extended because the model relates survival to stage at diagnosis. If detection occurred earlier but at the same cancer stage, then no survival benefit was gained. This is a conservative construction included to mitigate against positive biases such as lead time bias, length bias and over-diagnosis [21].

Baseline risk of death was based on life tables which were then adjusted for smoking and mortality rate due to lung cancer [22–24]. A Bayesian Markov Chain Monte Carlo analysis was conducted to calibrate mortality, prevalence, incidence and progression against the US NLST findings and the incidence of lung cancer in England [1, 4, 25, 26]. A log-normal distribution was assumed for preclinical incidence of lung cancer, and exponential distributions were assumed for the time to preclinical progression (from Stage IA to Stage IB, from Stage IB to Stage IIA, etc.) and the time to clinical presentation (according to the stage). It was assumed in the base case that there is no heterogeneity between patients in the rate at which their cancers progress or present, but this was tested in scenario analyses.

The probability of an individual responding to an initial questionnaire and the subsequent probability of participation given they met the eligibility criteria (46.5%) were estimated from the UKLS trial [5]. The probability of LDCT correctly identifying those with lung cancer (sensitivity) was 70.9%, estimated in the calibration exercise, and the probability of correctly identifying those without (specificity) was 62.4%, estimated from the UKLS trial [5]. Baseline utility values for smokers (in the absence of lung cancer or with preclinical disease) were estimated controlling for sex and age (0.753 for females and 0.782 for males for ages 75 to 84) [27], and stage-based utilities of smokers were estimated from the health-related quality of life (HRQoL) of lung cancer patients (stage II, 0.77; stage III, 0.77; stage IV, 0.76) [27, 28]. It was assumed that individuals with stage I cancers (mostly asymptomatic) would have the same utility as smokers without lung cancer. These utility values were used for men and women in the model regardless of the current smoking status (which was not modelled) and age. A small temporary disutility was applied for lung cancer screening itself (0.01 lasting for 2 weeks), and a more significant disutility following a false-positive result (0.063 lasting for 3 months) [29, 30].

Screening costs included the administration costs of self-assessment surveys, scoring, follow-up invite/decline of responder questionnaires, and the cost of LDCT examination(s) of programme joiners (this included a brief nurse assessment and the unit cost did not vary across first or subsequent screens) [31, 32]. Resources utilisation rate for diagnosis, treatment and follow-up were based on a retrospective 1-year cohort study of lung cancer patients at an English teaching hospital and were matched to disease stage at diagnosis [33]. Costs were applied for a maximum of 2 years post-diagnosis, with second year costs adjusted from the index year according to UK lung cancer resource patterns [34]. People who received a false-negative screen were zero treatment and follow-up cost until a true diagnosis of lung cancer, and false positives consumed resources as per cases in the UKLS trial [5]. End-of-life costs were included for lung cancer deaths [35]. Costs were inflated when needed to the adopted price year, 2016.

A summary of key input parameters are presented in Table 1, and key assumptions around modelling approach in Table 2; supplementary Tables S1 and S2 provide unabridged details. ENaBL is publicly available, accessed through the Open Research Exeter repository [37].

**Table 1:** Key input parameters of the model

Description	Base case value	Source
Number of smokers aged 55–80	13,000,000	Office of National Statistics [36] Health survey for England [27]
Probability someone responds to the initial invite and returns the questionnaire	0.307	UKLS trial [6]
Probability someone joins screening programme given they are eligible	0.465	UKLS trial [6]
Sensitivity of low-dose CT test for lung cancer	0.709	UKLS trial [6]
Specificity of low-dose CT test for lung cancer	0.624	UKLS trial [6]
Utility of male smoker in the UK general population/occult lung cancer	0.7816	Health Survey for England [27]
Utility of female smoker in the UK general population/occult lung cancer	0.7531	Health Survey for England [27]
Disutility associated with a false-positive screen	− 0.063	Mazzone et al*.* [30]
Disutility associated with anxiety of a screening event	− 0.010	NELSON trial [29]
Duration of disutility from false-positive screen	3 months	Assumption
Duration of disutility from screening anxiety	2 weeks	Assumption
Cost of low-dose CT scan	£98.80	NHS Reference costs 2015/2016 [31]

**Table 2:** Key assumptions of the model

Changes in smoking behaviour are not modelled.	
Programme uptake will be similar in real life to in the UKLS trial.	
There is full concordance with screening programme (i.e. no missed appointments)	
Health-related quality of life similar for preclinical and diagnosed lung cancer (stratified by stage).	
Health-related quality of life similar for clinically presenting and screen-detected lung cancer of the same stage.	
Health-related quality of life for diagnosed lung cancer is constant until death.	
Natural history of lung cancers is similar across all included individuals.	
Lung cancers progress through stages in numerical order without skipping any stages.	
Sensitivity of LDCT is independent of patient and tumour characteristics.	
Lung cancer mortality: screening cannot be less effective than no screening.	
Mortality from preclinical lung cancer assumed to be negligible.	
Lung cancer incidence in participating population similar to incidence in general smoking (current and former) population.	
Survival in participating population similar to survival in general population (stratified by stage).	
Incidental findings not modelled.	
True-positive results lead to immediate diagnosis and treatment.	
False-positive and indeterminate results are treated equivalently.	
Non-attendance of screening was not explicitly modelled	
Additional cancers caused by radiation exposure not modelled.	
Risk prediction is dependent only on prevalence of occult lung cancer or short-term incidence (within 3 years).	

Commentary on the impact of these assumptions and the outcome of testing alternative assumptions can be found in Supplementary Table S1.

## Results

### Cost-effectiveness

There were four screening strategies that could be cost-effective at different values of the cost-effectiveness threshold, and these form the cost-effectiveness frontier on the cost-effectiveness plane (Table 3). However, none of the screening strategies would be considered cost-effective versus no screening at a threshold of £20,000 per QALY. At a threshold of £30,000 per QALY, S-60-75-3% (screening programme design ‘S’; participation age range ‘60–75’ years; predicted lung cancer risk of ‘≥ 3%’) and S-55-75-3% would be cost-effective versus no screening (ICERs £28,169 and £28,784 per QALY gained, respectively).

**Table 3:** Base case results of screening strategies forming the cost-effectiveness frontier (per person)

Strategy	Change in 5-year lung cancer survival	Costs	QALYs	ICER (versus current/no screening)	ICER (versus previous)
No screening		£1103	8.50215		
S-60-75-3%^a^	+ 16.1%	£1126	8.50297	£28,169	£28,169
S-55-75-3%	+ 16.4%	£1129	8.50306	£28,784	£35,453
S-55-80-3%	+ 16.1%	£1135	8.50319	£30,821	£44,087
T-55-80-3%	+ 21.0%	£1151	8.50337	£40,034	£95,292

^a^In a fully incremental analysis, only S-60-75-3% would be cost-effective at a threshold of £30,000 per QALY gained. *ICER* incremental cost-effectiveness ratio, *QALY* quality-adjusted life year, *S* single one-off screen design, *T* triple-screen design. Strategy nomenclature: X-XX-XX-X% = screening programme design type-minimum entry age-maximum entry age-minimum lung cancer risk threshold

**Fig. 2:**
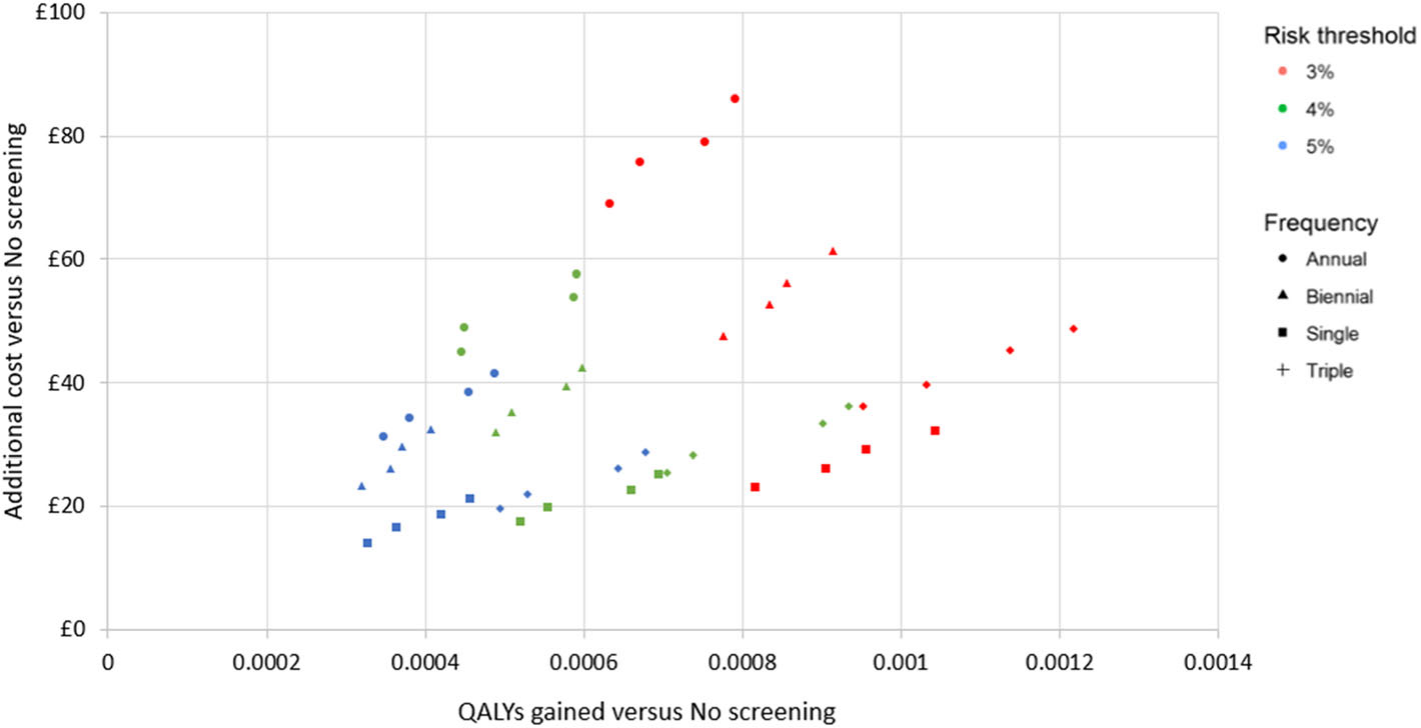
Cost-effectiveness plane for base case per person results across all strategies. QALY, quality-adjusted life year

**Fig. 3:**
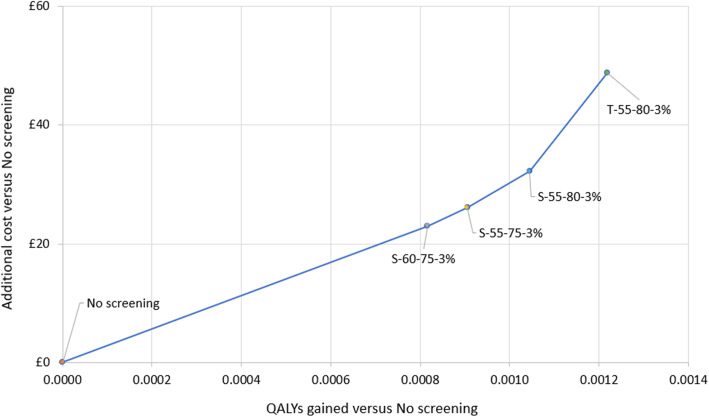
Cost-effectiveness plane for base case per person results of frontier strategies. QALY, quality-adjusted life year; S, single one-off screen design; T, triple-screen design. Strategy nomenclature: X-XX-XX-X% = screening programme design type-minimum entry age-maximum entry age-minimum lung cancer risk threshold

All strategies were predicted to lead to health benefits, ranging from 0.0003 to 0.0012 QALYs gained per person in the higher risk target population, including non-participating invitees. Health gains in programme participants were more significant, ranging from 1.2 to 4.0% of the population. Individuals participating in the S-60-75-3% screening programme were predicted to gain an average 0.054 life years (≈ 3 weeks) or 0.027 discounted QALYs versus no screening, and mortality from lung cancer was on average delayed 0.16 years (≈ 8 weeks). Figure 2 illustrates incremental costs and QALYs for each strategy on the cost-effectiveness plane. Figure 3 presents only those which form the cost-effectiveness frontier, i.e., those that provide the maximum net monetary benefit for at least one choice of the cost-effectiveness threshold.

### Secondary health outcomes

Screening increased the probability of lung cancer being diagnosed in the early stages (I and II) versus later stages (III and IV). The average odds ratios of early diagnosis in programme designs were predicted to be 2.44 (S), 3.29 (T), 3.83 (B) and 5.62 (A). The forward-shift in diagnosis in the single-screen programmes was most significant for stage IA cancers, which were detected in 13% of participants versus 6% in no screening. Screening also led to an increase in lung cancer lifetime diagnoses, i.e., what would be considered over-diagnosis. The average relative risk of a lung cancer diagnosis was 1.11 (S), 1.15 (T), 1.18 (B) and 1.20 (A), with screen detections in 47.7% (S), 64.2% (T), 72.1% (B) and 80.6% (A) of lung cancer diagnoses. Lung cancer diagnoses per 100,000 participants were on average 19,200 (S), 19,700 (T), 20,300 (B) and 20,600 (A), compared to 17,200 for no screening. Interval cancers—those presenting between screens—accounted for 3.9% (T), 11.8% (B) and 5.6% (A) of diagnoses in multiple-screen programmes. There was an average of 1.00 (S), 2.68 (T), 4.55 (B) and 8.03 (A) LDCT screens per participant, with 0.32 (S), 0.93 (T), 1.60 (B) and 2.96 (A) false positive or indeterminate results per participant, respectively. Reduction in risk of lung cancer mortality versus no screening ranged from 2.9 to 8.7% for participants. The average reduction in mortality due to lung cancer was 4.2% (S), 4.4% (T), 5.2% (B) and 7.7% (A), and five-year survival was predicted to be 20.3% (S), 26.2% (T), 29.1% (B) and 32.3% (A), compared to 4.7% for no screening. Lung cancer deaths per 100,000 participants were on average 15,200 (S), 15,100 (T), 15,000 (B) and 14,600 (A), compared to 15,800 for no screening. Detailed clinical outcomes for screening strategies on the cost-effectiveness frontier are presented in Table 4*.*

**Table 4:** Detailed clinical outcomes and costs, for participants of strategies on the cost-effectiveness frontier

Screening programme strategy	No screening	S-60-75-3%	S-55-75-3%	S-55-80-3%	T-55-80-3%
*Mean outcomes per participant (versus no screening)*					
Number of screens		1.00	1.00	1.00	2.70
Number of false positives		0.33	0.33	0.33	0.95
Lead time (years)		0.2995	0.2992	0.2952	0.3946
Life years gained (years)		0.0537	0.0568	0.0524	0.0762
QALYs		0.0008	0.0009	0.001	0.0012
Change in lung cancer mortality		− 0.80%	− 0.45%	− 0.43%	− 0.64%
Change in 5-year lung cancer survival		+ 16.1%	+ 16.4%	+ 16.1%	+ 21.0%
Change in survival with lung cancer (years)		+ 1.87	+ 1.89	+ 1.85	+ 2.44
Change in age at lung cancer diagnosis (years)		− 1.7	− 1.69	− 1.62	− 2.03
Change in age at death from any cause (years)		+ 0.05	+ 0.06	+ 0.05	+ 0.08
Change in age at death from lung cancer (years)		+ 0.16	+ 0.2	+ 0.23	+ 0.41
*Outcomes per 100,000 participants*					
Proportion of diagnoses arising from screening		44.4%	44.3%	47.1%	62.5%
Number of screen-detected cases		1710	1785	2335	3185
Number of interval cancers		0	0	0	215
Additional lung cancer diagnoses		295	300	450	590
Lung cancer deaths averted		170	100	120	180
Life years gained		5367	5677	5242	7617
*Costs for each participant (£, versus no screening)*					
LDCT screening		104	104	104	275
Lung cancer care		1458	1445	1469	1724
End-of-life		534	530	515	505
Total		2097	2080	2088	2504
*Population of 13 million smokers aged 55–80 years (lifetime costs, £ million)*					
Screening administration	0	80.16	110.97	118.66	118.66
LDCT screening	0	41.42	43.53	54.06	142.48
Lung cancer care	9355	9540	9547	9610	9742
End-of-life	4979	4972	4971	4970	4965
Total	14,334	14,633	14,673	14,753	14,968
Additional vs no screening		299.1	338.8	418.5	634.2

*LDCT* low-dose computed tomography, *QALY* quality-adjusted life year, *S* single one-off screen design, *T* triple-screen design. Strategy nomenclature: X-XX-XX-X% = screening programme design type-minimum entry age-maximum entry age-minimum lung cancer risk threshold

### Resources and costs

The costs per participant for LDCT screening and subsequent lung cancer care increased in accordance with the frequency of screening, up to £690 and £1118, respectively, as incrementally more diagnoses are made and more resources are consumed. The cost of end-of-life care decreased slightly as survival increased with frequency of screening, but if there are savings in the cost of treatment from earlier detection (not explicitly explored) then these are outweighed by the additional cost of over-diagnoses. Taking a relevant population of 13 million smokers aged 55 to 80 years old, the programmes are predicted to lead to population lifetime cost increases of £299 million to £634 million. The direct marginal cost of running a screening programme makes up less than half of this increased cost, with the rest being due to lung cancer care.

### Additional analyses

A secondary analysis of cost-effectiveness when varying screening frequency in fixed populations found that annual and biennial screening were dominated by triple screening in all populations, which always gave the most QALYs. Optimisation analysis of age limits and predicted risk, using net monetary benefit (willingness-to-pay £20,000 per QALY), identified a tentative target population, for the single-screen design, of age range 64 to 67 years and a minimum risk threshold of ≥ 2%, producing an ICER versus no screening of £13,361 per QALY gained.

### Uncertainty

Internal model validity was tested with univariate, scenario and probabilistic analyses. Univariate sensitivity analysis (supplementary Figure S1) found four of the five most influential input parameters were related to the natural history for smokers, the other was the cost of LDCT. The specificity of screening appears to be more influential than the sensitivity, but in both cases improved diagnostic performance leads to improved cost-effectiveness. Likewise, the performance level of risk prediction positively affects cost-effectiveness. In a scenario analysis, of the 17 alternatives explored, cost-effectiveness at £20,000 per QALY was achieved only when false-positive and indeterminate results were attributed nil effect on HRQoL, or when discounting of future costs and benefits was removed. Probabilistic sensitivity analysis (PSA) estimates compared well with deterministic estimates although the cost-effectiveness frontier moved to include only S-60-80-3% and S-55-75-3%, with the multiple screening strategy T-55-80-3% displaced. Cost-effectiveness acceptability curves in the PSA analysis showed that no screening had the highest probability of being cost-effective for thresholds up to £50,000 per QALY, although S-60-75-3% and S-60-80-3% are expected to be cost-effective at thresholds below £50,000 per QALY [38].

## Discussion

The lung cancer screening programmes simulated here are predicted to lead to health benefits for participants compared to no screening. We estimate a reduction in mortality from lung cancer ranging from 4.2 to 7.7%, depending on screening frequency, but find increased lung cancer diagnoses (including indolent cases), and increased lung cancer costs. In the base case analysis, it is predicted that when using a cost-effectiveness threshold of £20,000 per QALY, none of the programmes would be considered a cost-effective use of limited NHS resources versus the current UK strategy of no screening. At a higher cost-effectiveness threshold of £30,000 per QALY a single-screen offered to people aged 60 to 75 years with a predicted risk of lung cancer of at least 3% could be cost-effective. The PSA showed that when the threshold increased from £20,000 to £30,000 per QALY, the probability of no screening being the most cost-effective option decreased from 70 to 50%. However, at £30,000 per QALY, there are a number of LDCT screening strategies which could potentially be cost-effective, and therefore the probability of any one strategy being optimally cost-effective is low. Furthermore, simulation of this complexity is inherently uncertain.

One-way sensitivity analyses showed that a 10% variation in any single parameter is unlikely to result in LDCT screening being cost-effective at £20,000 per QALY and that ICERs were most sensitive to the modelling of the natural history of lung cancer, the cost of treating lung cancer and the cost of LDCT scans. Scenario analyses demonstrated that the impact of false-positive and indeterminate screening results on HRQoL was important in determining cost-effectiveness, as was the discount rate of future costs and benefits. While anxiety and distress from screening results may be studied, as well as potentially affected by a variety of interventions, the adjustment to decrease the value of future costs and benefits is applied broadly and similarly across health technology assessment. Since the health effects of lung cancer screening do not lag significantly behind the costs, because survival is generally poor and benefits are accrued relatively soon, there is no good case to deviate from standard approaches [39].

We believe ENaBL is the first economic evaluation of lung cancer screening to include a risk prediction component with a variable threshold, although risk proxies in the form of smoking histories have been used [40]. Further, this is the first UK-based model to adjust for over-diagnosis, the phenomenon in screening programmes whereby nodules/tumours which would not have been clinically significant during the patient’s life are detected. This has been estimated to be 18% of screen-detected lung cancers relative to chest X-ray screening or 31% relative to no screening [41]. The model results are driven substantially by the natural history model, which allows for the evaluation of multiple hypothetical screening programmes which have not been evaluated in clinical trials. The natural history component is based on high-quality evidence from the large US NLST RCT and UK national sources [1, 25]. Previous UK-based models have been based on ELCAP, a much smaller study [42]. This publicly available model predicts an impact on lung cancer mortality of − 4.2 to − 7.7% depending on the frequency of screening. This is in good agreement with the estimated 5% reduction in lung cancer mortality estimated by our associated network meta-analysis, providing some confidence in this modelled endpoint [15]. By using the discrete event simulation framework, there has been no need to artificially restrict the model states or distributions for event times or to consider a homogeneous cohort. There were few sensitivity analyses in which lung cancer screening became cost-effective at a threshold of £20,000 per QALY, which suggests some amount of robustness in the findings, although a number of key assumptions are not explored (e.g. no change in smoking behaviour, and impact of incidental findings). The model does not take the impact of screening on mortality as an input but produces it as an output resulting from the natural history model and the programme design. This helps internal validity and provides flexibility, so if additional mortality benefit needs to be incorporated, if it is demonstrated in future trials, then new assumptions and parameters will need to be introduced. Indeed, the current model predicts that the cost-effectiveness of screening is closely linked to the relative risk of lung cancer mortality: a relative risk of 0.935 in single-screen individuals aged 60–75 years with ≥ 3% risk of lung cancer would become cost-effective at £20,000 per QALY (although this is based on extrapolation and is therefore subject to significant uncertainty).

The costs of lung cancer have been estimated from a single English teaching hospital and therefore may not be fully generalisable to the whole of the UK at present, due to possible variation in clinical practice or use of technologies, and any significant changes in drug acquisition prices. This economic evaluation does not include a cost to identify target individuals from GP records, which would be non-zero but nominal. It takes the invitation response rate observed in UKLS, but a sample of individuals selected for trial may be biassed towards participation [5]. The economic evaluation currently assumes that the stage of lung cancer is relevant only to survival, it does not consider the relationship between the stage of lung cancer and the performance of LDCT, relevant because the identification of small nodules in early stage lung cancer may be more challenging [43]. Also, the model does not consider whether lung cancer type or location affects performance of screening, costs or survival.

Estimates of cost-effectiveness of screening across other health conditions are varied and, as in this case, are often complex and sensitive. Research for the UK National Screening Committee found that strategies for both bowel cancer (one-off faecal immunochemical test) and cervical cancer (primary HPV screen) are more effective and less costly than no screening [44, 45]. However, screening for ovarian cancer (multimodal) is either highly cost-effective versus no screening or dominated by no screening, depending on the chosen time horizon [46].

## Conclusion

Evidence from ENaBL suggests that LDCT screening for lung cancer may not be cost-effective, depending on the cost-effectiveness threshold used [47]. Thresholds of £20,000 to £30,000 per QALY are commonly used in the UK, and screening is estimated to be cost-effective with the higher threshold, but not with the lower. This evaluation suggests that screening would result in a reduction in lung cancer mortality, but also an increase in lung cancer diagnoses, and additional costs. One screening strategy that was investigated provided a ratio of additional costs to benefits that was at the upper limit of what would conventionally be considered cost-effective in the UK, while other screening strategies were outside the normal range of cost-effectiveness. Screening strategies with annual or biennial scans are not expected to be cost-effective regardless of the amount one is willing to pay for benefits. Early results from the NELSON trial appear to support the positive mortality benefits demonstrated by the NLST but may also show our finding to be conservative [48]. An extension of this economic evaluation—which considers multiple programme designs and adjusts for over-diagnosis bias—to include the full findings of the NELSON trial would significantly enhance the evidence base for decision-makers wishing to consider the cost-effectiveness of lung cancer screening in the UK.

## Supplementary Information

**Additional file 1.** Supplementary Table S1: Key assumptions in the model and their likely impact. Supplementary Table S2: Full parameter set for the model. Supplementary Figure S1. Tornado diagram for univariate sensitivity analysis.

## Data Availability

The mathematical model (ENaBL) supporting the conclusions of this article is available in the Open Research Exeter repository (http://hdl.handle.net/10871/33609) under a Creative Commons Attribution-NonCommercial-NoDerivatives 4.0 International Licence (CC BY-NC-ND 4.0) for the purposes of transparency, reproducibility and education. It is complete with the base case simulation data but simulation data from other analyses have not been retained by the authors.
