# Supplementary material for: Lung cancer screening by low-dose computed tomography: a cost-effectiveness analysis of alternative programmes in the UK using a newly developed natural history-based economic model
Source: Diagn Progn Res. 2020 Dec 2;4:20. doi: 10.1186/s41512-020-00087-y (PMC7709236; doi:10.1186/s41512-020-00087-y)
Supplement: Supplementary file 1 — Additional file 1. Supplementary Table S1: Key assumptions in the model and their likely impact. Supplementary Table S2: Full parameter set for the model. Supplementary Figure S1. Tornado diagram for univariate sensitivity analysis. [file 41512_2020_87_MOESM1_ESM.docx]

Supplementary Table S1: Key assumptions in the model and their likely impact

| Assumption | Anticipated impact (on cost-effectiveness of screening versus no screening versus likely true cost-effectiveness) | Outcome of scenario or sensitivity analyses |
| --- | --- | --- |
| Changes in smoking behaviour are not modelled | Possibly worsened; screening may encourage some to quit smoking, but evidence is mixed | Not conducted |
| Uptake will be similar in real life to in UKLS trial | Unclear; on the one hand trials tend to recruit healthier volunteers, but on the other hand invitations to participate in a trial contain substantially different information to invitations to participate in a screening programme | When the uptake of the screening was halved, S–60–75–3% was dominated and there were three screening strategies on the cost-effectiveness frontier: S–60–80–3% (£41,040 per QALY), T–60–80–3% (£97,030 per QALY) and B–60–80–3% (£263,700 per QALY).  When uptake of screening was doubled, S–60–75–3% was dominated and there were three screening strategies on the cost-effectiveness frontier: T–60–75–3% (£49,409 per QALY), T–60–80–3% (£84,823 per QALY) and T–55–80–3% (£382,200 per QALY). |
| Full concordance with screening programme (i.e., no missed appointments) | Improved; missed appointments lead to wasted resources and missed opportunities for patients to benefit from screening | Not conducted |
| Health-related quality of life similar for preclinical and diagnosed lung cancer (stratified by stage) | Unclear; a diagnosis of lung cancer may lead to heightened anxiety, and treatments for lung cancer may lead to reduced health-related quality of life, however, with a diagnosis patients may also receive better support to manage their symptoms | Not conducted |
| Health-related quality of life similar for clinically presenting and screen-detected lung cancer of the same stage | Unclear | Not conducted |
| Health-related quality of life for diagnosed lung cancer is constant until death | Worsened; one would expect health-related quality of life to diminish over time as the disease progresses despite treatment, and this would be particularly acute in those dying from lung cancer (and there are more of these without screening) | Not conducted |
| Natural history of lung cancers is similar across all included individuals | Improved; length bias and over-diagnosis are not fully addressed in the base case analysis, both of these phenomena undermine effectiveness | The natural history model was recalibrated assuming heterogeneity between patients in the rate of lung cancer progression. This also affected the estimated sensitivity of LDCT screening (increasing it substantially). In this scenario, S–60–75–3% was dominated and there was one screening strategy on the cost effectiveness frontier: S–60–80–5% (£167,136 per QALY). |
| Lung cancers progress through stages in numerical order without skipping any stages | Unclear; if lung cancers do skip stages in significant proportion then the natural history model calibrated to NLST data may not be appropriate | Not conducted |
| Sensitivity of low-dose CT independent of patient and tumour characteristics | Unclear; sensitivity may be expected to be worse for earliest stage cancers (who could potentially benefit the most from screening), but this would also have a significant impact on over-diagnosis | Not conducted |
| Lung cancer mortality methodology | Unclear; the methodology establishes a lower bound on effectiveness (so that screening cannot be less effective than no screening), but it is possible that survival is underestimated when a stage shift is achieved, since there is some evidence (though at high risk of bias) that screen-detected cancers have improved survival versus non-screen-detected cancers | Partially. There are scenario analyses where the impact on mortality is eliminated or attenuated, but no scenario analysis where the impact on mortality is strengthened.  When the survival benefit from early detection was eliminated (i.e. the survival is extended only by the lead time because of screening) all screening strategies were dominated by no screening. When the survival benefit from early detection was halved, S–60–75–3% was dominated and there were two strategies on the cost-effectiveness frontier: S–60–75–5% (£74,157 per QALY) and T–60–75–5% (£121,200 per QALY). |
| Mortality from preclinical lung cancer assumed to be negligible | Worsened; if there is significant mortality from preclinical (occult) lung cancer in the population, then screening would potentially be able to reduce this | Not conducted |
| Lung cancer incidence in participating population similar to incidence in general smoking (current and former) population | Possibly improved; respondents are likely to be healthier than general smoking population and therefore may have reduced incidence, however, the use of a risk prediction model should substantially mitigate this | Refer to the univariate sensitivity analysis |
| Survival in participating population similar to survival in general population (stratified by stage) | Improved; it is more likely that a participating population would be healthier and less deprived than the general population of smokers and would therefore have improved survival, therefore potentially benefitting less from screening | Refer to the univariate sensitivity analysis |
| Incidental findings not modelled | Unclear; incidental findings may be of clinical value (i.e., it may be possible to offer treatment or management which improves patient outcomes) but may also significantly increase costs | Not conducted |
| True positive results lead to immediate diagnosis and treatment | Improved; there is expected to be a delay between screening and diagnosis, during which lung cancer could progress further, so reducing the benefit of screen detection | Not conducted |
| False positive and indeterminate results are treated equivalently | Unclear; indeterminate results typically result in less intensive follow-up than false positive results, but the model assumes a weighted average of these results according to the UKLS trial | Not conducted |
| Non-attendance of screening was not explicitly modelled | Unclear; NHS reference costs include costs of missed appointments as overheads within unit costs, but it is not clear whether the unit cost chosen will include a representative overhead for non-attendance in a hypothetical screening programme | Refer to the univariate sensitivity analysis (unit cost of LDCT) |
| Additional cancers caused by radiation exposure not modelled | Improved; additional cancers would lead to increased costs and decreased QALYs | Not conducted |
| Risk prediction is dependent only on prevalence of occult lung cancer or short-term incidence (within three years) | Worsened (especially for annual and biennial strategies); in the model the value of risk prediction is limited to the first three years, such that individuals who would develop lung cancer more than three years later have no higher predicted risk on average than individuals who would not develop lung cancer beyond three years | Not conducted |

Supplementary Table S2: Full parameter set for the model

| Label | Description | Base case value | PSA |
| --- | --- | --- | --- |
| Population |  |  |  |
| pop_size | Number of smokers aged 55-80 | 13,000,000 | Not varied |
| p_male | Proportion of those receiving risk prediction who are men | 0.482 | Beta(29393,31609) |
| pop_age_mean | Population mean age | 61.939 | N(61.939,0.048) |
| pop_age_sd | Population standard error | 9.000 | N(8.999,0.062) |
| pop_age_LL | Quantile for lower age limit of whole population and lower boundary of age at entry distribution | 0.220 | Not varied |
| pop_age_UL | Quantile for upper age limit of whole population and upper boundary of age at entry distribution | 0.978 | Not varied |
| Programme uptake |  |  |  |
| p_respond | Probability someone responds to the initial invite and returns the questionnaire | 0.307 | Beta(75958,171396) |
| p_join | Probability someone joins screening programme given they are eligible | 0.465 | Beta(4061,4668) |
| Natural history of disease |  |  |  |
| mu_AB | Lognormal parameter (location) for pre-clinical incidence of lung cancer | 4.7470 | Multivariate normal 1 |
| delta_mu_AB_F | Coefficient for women for lognormal parameter (location) for pre-clinical incidence of lung cancer | 0.0358 | Multivariate normal 1 |
| sigma_AB | Lognormal parameter (shape) for pre-clinical incidence of lung cancer | 0.3635 | Multivariate normal 1 |
| ln_lambda_pIA_pIB | Log rate of pre-clinical progression from stage Ia to Ib | 0.0035 | Multivariate normal 1 |
| ln_lambda_pIB_pIIA | Log rate of pre-clinical progression from stage Ib to IIa | 1.6451 | Multivariate normal 1 |
| ln_lambda_pIIA_pIIB | Log rate of pre-clinical progression from stage IIa to IIb | 1.8006 | Multivariate normal 1 |
| ln_lambda_pIIB_pIIIA | Log rate of pre-clinical progression from stage IIb to IIIa | 1.6258 | Multivariate normal 1 |
| ln_lambda_pIIIA_pIIIB | Log rate of pre-clinical progression from stage IIIa to IIIb | 1.0797 | Multivariate normal 1 |
| ln_lambda_pIIIB_pIV | Log rate of pre-clinical progression from stage IIIb to IV | 2.0803 | Multivariate normal 1 |
| ln_lambda_pIA_cIA | Log rate of clinical presentation at stage Ia | -2.4828 | Multivariate normal 1 |
| ln_lambda_pIB_cIB | Log rate of clinical presentation at stage Ib | -1.8726 | Multivariate normal 1 |
| ln_lambda_pIIA_cIIA | Log rate of clinical presentation at stage IIa | -1.6507 | Multivariate normal 1 |
| ln_lambda_pIIB_cIIB | Log rate of clinical presentation at stage IIb | -2.1362 | Multivariate normal 1 |
| ln_lambda_pIIIA_cIIIA | Log rate of clinical presentation at stage IIIa | -1.4088 | Multivariate normal 1 |
| ln_lambda_pIIIB_cIIIB | Log rate of clinical presentation at stage IIIb | -0.8811 | Multivariate normal 1 |
| ln_lambda_pIV_cIV | Log rate of clinical presentation at stage IV | -1.4027 | Multivariate normal 1 |
| Survival from diagnosis |  |  |  |
| lambda_lcs_Ia | Lambda constant for survival if diagnosed and treated from stage Ia | 0.214 | N(0.214,0.011) |
| lambda_lcs_Ib | Lambda constant for survival if diagnosed and treated from stage Ib | 0.274 | N(0.274,0.014) |
| lambda_lcs_IIa | Lambda constant for survival if diagnosed and treated from stage IIa | 0.330 | N(0.33;0.016) |
| lambda_lcs_IIb | Lambda constant for survival if diagnosed and treated from stage IIb | 0.475 | N(0.475,0.024) |
| lambda_lcs_IIIa | Lambda constant for survival if diagnosed and treated from stage IIIa | 0.588 | N(0.588,0.029) |
| lambda_lcs_IIIb | Lambda constant for survival if diagnosed and treated from stage IIIb | 0.909 | N(0.909,0.045) |
| lambda_lcs_IV | Lambda constant for survival if diagnosed and treated from stage IV | 1.423 | N(1.423,0.071) |
| gamma_lcs_all_stages | Gamma constant for survival if diagnosed and treated at any stage | 0.676 | N(0.676,0.034) |
| lambda_ocm_F | Lambda parameter (Gompertz distribution) for other cause mortality in women | 0.00019 | N(0.000195,0.00001) |
| gamma_ocm_F | Gamma parameter for above | 0.1018 | N(0.102,0.005) |
| lambda_ocm_M | Lambda as above for men | 0.00059 | N(0.00059,0.00003) |
| gamma_ocm_M | Gamma as above for men | 0.0917 | N(0.092,0.005) |
| Risk prediction |  |  |  |
| risk_age | Risk prediction coefficient for age (years) | 0.08985 | N(0.090,0.00038) |
| risk_male | Risk prediction coefficient for male sex | 0.30562 | N(0.306,0.005) |
| risk_smoker | Risk prediction coefficient for current/former smoker (vs never smoker) | 1.45929 | N(1.459,0.005) |
| risk_lungcancer | Risk prediction coefficient for lung cancer (at baseline or within 3 years) | 0.33488 | N(0.335,0.152) |
| risk_intercept | Risk prediction intercept | -11.39758 | N(-11.398,0.024) |
| risk_SD | Risk prediction standard deviation (error term) | 0.62920 | Gamma(25,0.025) |
| Screening effectiveness |  |  |  |
| sens_LDCT | Sensitivity of low dose CT test for lung cancer | 0.709 | Multivariate normal 1 |
| spec_LDCT | Specificity of low dose CT test for lung cancer | 0.624 | Beta(740,445) |
| mu_ind_scrn_delay | Mean time to index screening examination | -2.823 | Multivariate normal 2 |
| sig_ind_scrn_delay | SE time to index screening exam | 0.820 | Multivariate normal 2 |
| Quality of life |  |  |  |
| u_base_male | Utility of male smoker in the UK general population/occult lung cancer | 0.7816 | N(0.782,0.012) |
| u_base_female | Utility of female smoker in the UK general population/occult lung cancer | 0.7531 | N(0.753,0.11) |
| u_dis_sII | Disutility of second stage cancer versus first stage | -0.04 | N(-0.04,0.013) |
| u_dis_sIII | Disutility of third stage cancer versus first stage | -0.04 | N(-0.04,0.009) |
| u_dis_sIV | Disutility of fourth stage cancer versus first stage | -0.05 | N(-0.05,0.01) |
| u_dis_fp | Disutility associated with a false positive screen | -0.063 | N(-0.063,0.028) |
| u_dis_scr_anx | Disutility associated with anxiety of a screening event | -0.010 | N(-0.01,0.007) |
| t_dis_fp | Duration of disutility from false positive screen | 3.00 | Gamma(4,0.75) |
| t_dis_scrn_anx | Duration of disutility from screening anxiety | 2.00 | Gamma(4,0.5) |
| Costs |  |  |  |
| c_invite | Cost of initial invite and questionnaire | £2.90 | Gamma(25,0.116) |
| c_score | Cost of scoring questionnaire and risk stratification | £18.54 | Gamma(25,0.742) |
| c_letter | Cost of follow-up letter and (if applicable) LDCT appointment | £1.74 | Gamma(25,0.07) |
| c_gp_ref | Cost of GP consultations leading to lung cancer referral | £72.00 | Gamma(25,2.88) |
| c_LDCT | Cost of low dose CT scan | £98.80 | Gamma(59.126,1.671) |
| c_scrn_nurse | Cost of nurse-led screening consultation | £6.25 | Gamma(25,0.25) |
| c_false_pos | Cost of resourcing following a false positive screen | £184.63 | Gamma(25,7.385) |
| c_eol_lung | Cost of end-of-life care for lung cancer patient | £4,589.04 | Gamma(3.329,1378) |
| c_rdtf_sIa_ini | Cost of initial diagnosis and treatment if dx stage Ia | £5,558.14 | Gamma(25,222) |
| c_rdtf_sIb_ini | Cost of initial diagnosis and treatment if dx stage Ib | £6,411.63 | Gamma(25,256) |
| c_rdtf_sIIa_ini | Cost of initial diagnosis and treatment if dx stage IIa | £7,279.07 | Gamma(25,291) |
| c_rdtf_sIIb_ini | Cost of initial diagnosis and treatment if dx stage IIb | £6,558.14 | Gamma(25,262) |
| c_rdtf_sIIIa_ini | Cost of initial diagnosis and treatment if dx stage IIIa | £6,511.63 | Gamma(25,260) |
| c_rdtf_sIIIb_ini | Cost of initial diagnosis and treatment if dx stage IIIb | £6,046.51 | Gamma(25,242) |
| c_rdtf_sIV_ini | Cost of initial diagnosis and treatment if dx stage IV | £5,441.86 | Gamma(25,218) |
| c_rdtf_sIa_rem_ind_yr | Cost of index year diagnosis, treatment and follow-up if dx stage Ia | £5,848.11 | Gamma(25,234) |
| c_rdtf_sIb_rem_ind_yr | Cost of index year diagnosis, treatment and follow-up if dx stage Ib | £5,359.21 | Gamma(25,214) |
| c_rdtf_sIIa_rem_ind_yr | Cost of index year diagnosis, treatment and follow-up if dx stage IIa | £5,637.60 | Gamma(25,226) |
| c_rdtf_sIIb_rem_ind_yr | Cost of index year diagnosis, treatment and follow-up if dx stage IIb | £6,514.78 | Gamma(25,262) |
| c_rdtf_sIIIa_rem_ind_yr | Cost of index year diagnosis, treatment and follow-up if dx stage IIIa | £5,415.46 | Gamma(25,217) |
| c_rdtf_sIIIb_rem_ind_yr | Cost of index year diagnosis, treatment and follow-up if dx stage IIIb | £4,318.07 | Gamma(25,173) |
| c_rdtf_sIV_rem_ind_yr | Cost of index year diagnosis, treatment and follow-up if dx stage IV | £2,787.31 | Gamma(25,111) |

Supplementary Figure S1. Tornado diagram for univariate sensitivity analysis.


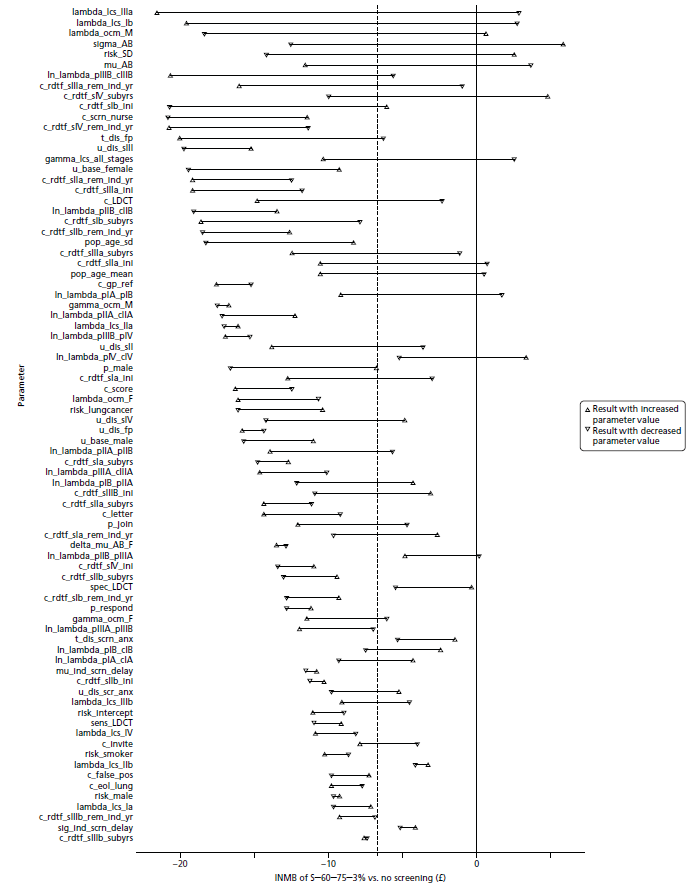


Parameters are sorted by descending range of INMB produced. Abbreviations: INMB, Incremental net monetary benefit.
